# Supplementary material for: Synergistic potential of lopinavir and azole combinational therapy against clinically important Aspergillus species
Source: PLoS One. 2024 Dec 2;19(12):e0314474. doi: 10.1371/journal.pone.0314474 (PMC11611203; doi:10.1371/journal.pone.0314474)
Supplement: Attachment — Submitted filename: comments.docx [file pone.0314474.s001.docx]

Please add additional comments for the authors:

**General comment:**

The paper by Nicolas Burns *et al.* is about the potential synergistic effect of the antiviral drug lopinavir (LPV) in combination with the azole (ITC and POS) against clinically important *Aspergillus* species. It evaluates the impact of this combination therapy on *A. fumigatus* strains and other clinically important *Aspergillus* species, as well as the potential mechanisms underlying the observed synergistic effects. Additionally, the study addresses the impact of the LPV/azole combination on the hyphal formation, a critical step for fungal dissemination. However, this manuscript exists several limitations. Firstly, the study acknowledges the limitations of LPV's synergistic activity with certain azole drugs, such as voriconazole, and the potential differential response observed between various azoles. Secondly, the potential toxicity of this combination probably exists. Furthermore, the study focus on *in vitro* combination treatment, and the amount of the strains are limited considering the variability between strains of the same species, and *in vivo* treatment is still unknown.

The manuscript requires some improvements; see below for detailed comments.

**Abstract:** In general, methods should be involved in this part.

**Introduction:** In this part, the reason for choosing LPV combination for treatment should be an independent part and be highlighted.

Line 68: “cyp51A”, use italic.

Line 71:” Non-Cyp51A ” change into “Non-*cyp51A* ”

Line 80-81: “we focused on LPV and antiviral agent as an enhancer of azoles against

*Aspergillus* species”. Here can be changed into “we focused on antiviral agent (LPV) as an enhancer of azoles against *Aspergillus* species”.

Line 77-94: Separate the introduction of LPV and your study content.

**Results:** In general, many parts belong to methods and discussion or conclusion, when describing the results, there is no need to repeat the methods. Subtitles should not be end with “.”. Figures when mentioned in context should keep identity. The detailed comments are as follows:

Line 97-102 : Here belongs to “Methods and Materials”.

Line 118-120 : “figure 1”, change into “Figure 1”, better put it in bracket.

Line 121-122: Subtitle remove the “.”.

Line 123-126: Here belongs to “Methods and Materials” as well. In addition, what’s the concentration of conidia?

Line 126: “figure 2”, the same as above

Line 136: “fig. 3”, keep identity in context.

Line 138-139: This is the discussion or conclusion.

Line 141-142: before “When”, add “.” to make the sentence completely.

Line 145-149: “figure”, same as above.

**Discussion**

This section requires some revision.

Line 155-156: “In recognition of this significant concern, the WHO has designated Aspergillosis as a critical priority pathogen”. Aspergillosis is a kind of disease, not a pathogen.

Line 169-176: Is there any literature that mentions about the link between these mutations and the effects of antiviral drugs in combination with azoles? What’s the difference compared with the previous study?

Line 179-181: Why does it work against *Candida* for the combination of LPV and VRC?

Line 184-185: “Our initial checkerboard data demonstrated that the co-application of LPV enhances azole activity against susceptible strains, as well as two CDC-resistant strains”, add “.” at the end of this sentence.

Line 189-196: This paragraph belongs to “Result”. Add some analysis and references or remove them.

Line 211-215: Concise this part.

Line 216-217: “While ITC and POS can be enhanced by efflux inhibition VRC demonstrated little increased efficacy.”, add “,” after “inhibition”.

Line 216-217: Why the combination of VRC showed different effect?

Line 222-225: Concise into one sentence.

Line 224: Do you think these toxic patches can cause potential damage to cells or humans when applied to clinical therapy?

**Conclusion :** Here need improvement and condense. Some expressions are not accurate. The study mainly focused on the treatment effects based on the combination of LPV and azoles *in vitro*. Therefore, there remains a long process from i*n vitro* to *in vivo* application. In addition, the potential damage after the combination of these drugs probably cause is still unknown.

Line 240-241: This is the reference, not your conclusion for your study.

**Method：**In general, which method you have referred to must be described and should contain more detailed information in this part. Please list the standard methods or protocols you have chosen for MICs. Also about data analysis, what method was used to analyse? Which software ?

Line 248-249: What is “CDC” and BEI? “table 1” use capital letter for first letter. And put it in bracket.

Line 256: Keep the tense identity.

Line 249-258: Make the format of reagents identity. For example, list the producer and place in the bracket.

Line 260-261: What’s protocol for MICs ?

Line 266-267: What are the published ranges?

Line 269-270: Why here use reference, do you mean that the isolates have been studied? And why you choose this isolate? Why not others or more?

Line 270: Why use this concentration of conidia and is that the final concentration?

Line 270-272: How can you choose the concentrations of different drugs? How do you know this concentration can work?

Line 272-277: Why are the time points setting of POS/ITC and VRC different?

Line 282-288: Did you have any reference for this method? Which strain was applied for this study?

Line 293-294: Why did you choose this concentration of the drugs?

**Figure 1**: Use different colors for different drugs in the control and tested groups. For example, Red/Purple/Pink contain for ITC/POS/VRC control group, while Blue/Green/Yellow for the LPV combination with ITC/POS/VRC, respectively.

Use a.b.c for sub-figures.

Abbreviations should be interpreted.

**Figure 2**: The subtitle should be removed in the figures, and it already showed in the legends. The legend should be clear and concise. Abbreviations should be interpreted.

**Figure 4:** How did you know the patches that were toxic?

**Table. 1.** About “Source & Characteristic & Resistance Mechanism”, you should add the reference if you did not test in this study, for example, the mutation mentioned in this table.
