# Supplementary material for: Synergistic potential of lopinavir and azole combinational therapy against clinically important Aspergillus species
Source: PLoS One. 2024 Dec 2;19(12):e0314474. doi: 10.1371/journal.pone.0314474 (PMC11611203; doi:10.1371/journal.pone.0314474)
Supplement: Attachment — Submitted filename: Response to Reviewers 032024.docx [file pone.0314474.s002.docx]

Reviewer's Responses to Questions

Reviewer #1: Please add additional comments for the authors:

General comment:
The paper by Nicolas Burns et al. is about the potential synergistic effect of the antiviral drug lopinavir (LPV) in combination with the azole (ITC and POS) against clinically important Aspergillus species. It evaluates the impact of this combination therapy on A. fumigatus strains and other clinically important Aspergillus species, as well as the potential mechanisms underlying the observed synergistic effects. Additionally, the study addresses the impact of the LPV/azole combination on the hyphal formation, a critical step for fungal dissemination. However, this manuscript exists several limitations. Firstly, the study acknowledges the limitations of LPV's synergistic activity with certain azole drugs, such as voriconazole, and the potential differential response observed between various azoles. Secondly, the potential toxicity of this combination probably exists. Furthermore, the study focus on in vitro combination treatment, and the amount of the strains are limited considering the variability between strains of the same species, and in vivo treatment is still unknown.
The manuscript requires some improvements; see below for detailed comments.

Abstract: In general, methods should be involved in this part.

We thank the reviewer for his comment, and as per their suggestion, we have incorporated a methodology section in the abstract.
Introduction: In this part, the reason for choosing LPV combination for treatment should be an independent part and be highlighted.

We thank the reviewer for the comment and have added the following:

“Our lab previously identified lopinavir (LPV) as a potent enhancer to azole antifungals against multi-drug resistant *Candida auris*. Expanding further on this, we focused on the antiviral agent (LPV) as an enhancer of azoles against *Aspergillus* species.”
Line 68: “cyp51A”, use italic.

We thank the reviewer for their comment and have corrected this.
Line 71:” Non-Cyp51A ” change into “Non-cyp51A ”

We thank the reviewer for their comment and have corrected this.
Line 80-81: “we focused on LPV and antiviral agent as an enhancer of azoles against
Aspergillus species”. Here can be changed into “we focused on antiviral agent (LPV) as an enhancer of azoles against Aspergillus species”.

We thank the reviewer for their comment and have amended the document as such:

Line 76-77 “we focused on an antiviral agent (LPV) as an enhancer of azoles against *Aspergillus* species”
Line 77-94: Separate the introduction of LPV and your study content.
We thank the reviewer for their comment and have broken the paragraph at line 80
Results: In general, many parts belong to methods and discussion or conclusion, when describing the results, there is no need to repeat the methods. Subtitles should not be end with “.”. Figures when mentioned in context should keep identity. The detailed comments are as follows:
Line 97-102 : Here belongs to “Methods and Materials”.

We thank the reviewer for their comment and have amended the paragraph to reflect only the results.

Lines 94-98 “To determine the MICs for ITC, POS, VRC and LPV against 27 different Aspergillus isolates, we perfromed three independent assays following CLSI protocol M38 (23). MICs for Itraconazole ranged from 0.25 to 64 µg/mL, while posaconazole and voriconazole ranged from 0.25 to 2 µg/mL (24). CLSI reference isolates were determined to be within established ranges (23, 25). Notably, five isolates displayed resistance to azoles, with MICs of 64, 1, and ~1 µg/mL for ITC, POS and VRC, respectively. LPV, on the other hand, did not exhibit antifungal activity up to 256 µg/mL (Table 1).”
Line 118-120 : “figure 1”, change into “Figure 1”, better put it in bracket.

We thank the reviewer for their comment and have corrected this.

Line 123-126: Here belongs to “Methods and Materials” as well. In addition, what’s the concentration of conidia?

We thank the reviewer for their comment and have amended the document. The concentration is 2.5x10^5.

Lines 119-121: “To investigate the effect of azole/LPV combination on the growth kinetics of A. fumigatus, we conducted a growth kinetics assay using A. fumigatus strain CDC #738. As expected LPV, POS, VRC and ITC alone did not have a significant impact on fungal growth and closely resembled the untreated control (Fig 2).”
Line 126: “figure 2”, the same as above

We thank the reviewer for their comment and have corrected this.
Line 136: “fig. 3”, keep identity in context.

We thank the reviewer for their comment and have corrected this.
Line 138-139: This is the discussion or conclusion.

We thank the reviewer for their comment and have moved the final sentence to the discussion.
Line 141-142: before “When”, add “.” to make the sentence completely.

We thank the reviewer for their comment and have corrected this.
Line 145-149: “figure”, same as above.

We thank the reviewer for their comment and have corrected this.

Discussion
This section requires some revision.
Line 155-156: “In recognition of this significant concern, the WHO has designated Aspergillosis as a critical priority pathogen”. Aspergillosis is a kind of disease, not a pathogen.

We thank the reviewer for their comment and have edited it to reflect the WHO’s report.

Line 151: “the WHO has designated Aspergillus fumigatus as a critical priority pathogen (27).”
Line 169-176: Is there any literature that mentions about the link between these mutations and the effects of antiviral drugs in combination with azoles? What’s the difference compared with the previous study?

We thank the reviewer for their comments. There is not much in the way of studies or reviews describing azole and antiviral interaction, as aspergillus infections are severely underreported there are likely instances of this occur but have yet to be documented. This study itself focuses on another critical priority pathogen, while the studies do share similarity in the mechanism, efflux assay, we would point out the difference in the usage of morphological images to describe the toxic patch formation further supporting the robustness of this combination.
Line 179-181: Why does it work against Candida for the combination of LPV and VRC?

We thank the reviewer for their comment. According to our previously published data (Eldesouky et al 2020, Salama et al 2023, Elgammal et al., 2023), LPV did not work synergistically with VRC against most of the tested *Candida* isolates which is consistent with the current study. These findings are also consistent with earlier reports indicating that itraconazole and posaconazole are less impacted by ERG11 mutations, which are known to cause substantial resistance to other azoles like fluconazole and voriconazole (Flowers et al., 2015, Rodrigues et al., 2018).

Line 184-185: “Our initial checkerboard data demonstrated that the co-application of LPV enhances azole activity against susceptible strains, as well as two CDC-resistant strains”, add “.” at the end of this sentence.

We thank the reviewer for their comment and have corrected this.
Line 189-196: This paragraph belongs to “Result”. Add some analysis and references or remove them.

We thank the reviewer for their comment and have removed this.
Line 211-215: Concise this part.

We thank the reviewer for their comment and have shortened the section

Line 197-198: “cells treated with LPV have an impressive retention rate of nearly 70% of Rhodamine-6G. This has been observed in other proposed efflux pump inhibitors (32).”
Line 216-217: “While ITC and POS can be enhanced by efflux inhibition VRC demonstrated little increased efficacy.”, add “,” after “inhibition”.

We thank the reviewer for their comment and have corrected this.
Line 216-217: Why the combination of VRC showed different effect?

We thank the reviewer for their question. According to the previously published data, itraconazole and posaconazole are less impacted by ERG11 mutations, which are known to cause substantial resistance to other azoles like fluconazole and voriconazole (Flowers et al., 2015, Rodrigues et al., 2018).

Line 222-225: Concise into one sentence.

We thank the reviewer for their comment and have edited it as such.

Lines 205-207: “Research findings suggest that azole antifungals exert their fungicidal activity in Aspergillus by inducing the creation of cell wall carbohydrate patches comprised of both toxic and nontoxic sterol intermediates, trigger the demise of the fungus (26).”
Line 224: Do you think these toxic patches can cause potential damage to cells or humans when applied to clinical therapy?

We thank the reviewer for the question. There are side-effects associated with taking azoles. However, for the toxic patches to form within a human, the azole would need to inhibit a synthesis pathway that is not present. Therfore, we don't expect to have similar toxic effects on human cells.

Conclusion : Here need improvement and condense. Some expressions are not accurate. The study mainly focused on the treatment effects based on the combination of LPV and azoles in vitro. Therefore, there remains a long process from in vitro to in vivo application. In addition, the potential damage after the combination of these drugs probably cause is still unknown.

Line 240-241: This is the reference, not your conclusion for your study.

We thank the reviewer for this comment and have relocated the sentence to lines 80-83.

Method：In general, which method you have referred to must be described and should contain more detailed information in this part. Please list the standard methods or protocols you have chosen for MICs. Also, about data analysis, what method was used to analyse? Which software?

We thank the reviewer for their questions. For the MIC assay, we used the protocol described in the CLSI M38 and added a brief description of the methodology to the materials and methods section. For data analysis from the MIC values were recorded in excel and averaged from three independent experiments.
Line 248-249: What is “CDC” and BEI? “table 1” use capital letter for first letter. And put it in bracket.

We thank the reviewer for their comment and have spelled out the company names and reformatted the word.
Line 256: Keep the tense identity.

We thank the reviewer for their comment and have corrected this.
Line 249-258: Make the format of reagents identity. For example, list the producer and place in the bracket.

We thank the reviewer for their comment and have modified it accordingly.
Line 260-261: What’s protocol for MICs ?

We thank the reviewer for their question, the protocol for MIC is a broth microdilution method and we have incorporated a brief description in the methodology.
Line 266-267: What are the published ranges?

We thank the reviewer for their question however these are values that are utilized to interpret the results (ref 39-41).
Line 269-270: Why here use reference, do you mean that the isolates have been studied? And why you choose this isolate? Why not others or more?

We thank the reviewer for their question and apologize for the confusion. The reference is for the growth kinetic assay that we then describe with the next sentence. This isolate was selected from our list as it responded well to the combinational therapies.
Line 270: Why use this concentration of conidia and is that the final concentration?

We thank the reviewer for their question, We used this inoculum size because it is the standard inoculum specified in the CLSI M38 protocol.
Line 270-272: How can you choose the concentrations of different drugs? How do you know this concentration can work?

We thank the reviewer for their question. We selected the concentrations of the tested drugs here based on the results obtained from the checkerboard assays which indicated potential activity for 48 hours.

Line 272-277: Why are the time points setting of POS/ITC and VRC different?

We thank the reviewer for this question. We realized that even though VRC was unresponsive with the combinations it was re-added last with less timepoints as it was expected to fail.
Line 282-288: Did you have any reference for this method? Which strain was applied for this study?

We thank the reviewer for their question. The references for this assay are 20, 32, 44 and 45 located in line 281. We have also corrected the omission of the strain.
Line 293-294: Why did you choose this concentration of the drugs?
We thank the reviewer for this question. As with the growth kinetics assay all values were chosen from the checkerboard as they demonstrated no observable growth at 48 hours.

Figure 1: Use different colors for different drugs in the control and tested groups. For example, Red/Purple/Pink contain for ITC/POS/VRC control group, while Blue/Green/Yellow for the LPV combination with ITC/POS/VRC, respectively.
Use a.b.c for sub-figures.
Abbreviations should be interpreted.

We thank the reviewer for their comments and have made the necessary changes.
Figure 2: The subtitle should be removed in the figures, and it already showed in the legends. The legend should be clear and concise. Abbreviations should be interpreted.

We thank the reviewer for their comments and have made the appropriate changes.

Figure 4: How did you know the patches that were toxic?

We thank the reviewer for this question. The patches were described as toxic by Geissel et al., as being hallmarks for effective azole therapy. This also was consistent with the inhibitory effect of the combinations (Reference 28).


Table. 1. About “Source & Characteristic & Resistance Mechanism”, you should add the reference if you did not test in this study, for example, the mutation mentioned in this table.

We thank the reviewer for this comment; the information within the table is derived from the data available in the vendors’ websites. Therefore, we included links to this information and emphasized its relevance in the materials and methods.

Reviewer #2: In this manuscript, the authors have studied the synergistic antifungal activity combinational treatment with lopinavir (LPV, an FDA approved antiviral drug) and azoles (antifungal drug) against pathogenic Aspergillus species. They demonstrate that LPV inhibits fungal efflux pump, thereby increasing the efficiency of azoles.

Comments:

1. It is an interesting as well as important study. However, the study reads as if the authors focused on specific activity reported earlier for LPV, i.e., inhibition of efflux pump. It will be interesting also to know whether LPV increases the permeability of fungus facilitating the entry of azoles to execute their function.

We thank the reviewer for highlighting the importance of the study. In this study, we confirmed that LPV can interfere with the fungal efflux pump machinery. However, we agree with the reviewer that this does not exclude the possibility of LPV having another mechanism of action. Conducting a separate and extensive mechanistic study in the future could help elucidate this further.

2. Methodology of efflux pump inhibition assay is unclear. After transferring fungal cells into opaque plates and adding glucose to initiate efflux pump, in which compartment (inside or outside fungal cells) the florescence was measured and how?

We thank the reviewer for their comment. The fluorescence intensity results from the accumulation of R6G inside the cells. In the presence of an efflux pump inhibitor the accumulation of R6G is enhanced upon the addition of glucose which is clearly represented by high fluorescence inside the cells (Srivastava and Ahmad 2020). The fluorescence intensity was measured in an opaque 96-well plate at 430/485 nm using a SpectraMax i3x microplate reader.

3. Figure 4, particularly 4F is blurred. Better image and with higher magnification should be provided.

We thank the reviewer for their comment. We have edited the image to increase clarity and to display the patches better.

4. Data on other Aspergillus species in the form of a figure should be included.

We thank the reviewer for their comment. Here, we specifically focused on Aspergillus fumigatus because it is the most common cause of human aspergillosis. That's why we included A. fumigatus in the figures. However, to investigate if our findings are applicable to other species, we added other Aspergillus species to the table..

Minor comments (typos):

1. Line 80: Candida should be in italics.

We thank the reviewer for their comment and have corrected this.
2. Line 120: Figure 1 should be inside parenthesis.

We thank the reviewer for their comment and have corrected this.
3. Line 142: Put a full-stop after “induced by azoles”.

We thank the reviewer for their comment and have corrected this.
4. Paragraph 141 to 152: all figures (figure 4a to figure 4f) should be with parenthesis.

We thank the reviewer for their comment and have corrected this.
5. Line 185: There should be a full-stop after CDC-resistant strains.

We thank the reviewer for their comment and have corrected this.
6. Line 218: Leave a space between activity and reference numbers.

We thank the reviewer for their comment and have corrected this.
7. Line 261: Leave a space between assay and reference numbers.

We thank the reviewer for their comment and have corrected this.

Reviewer #3: Lines 88-94: these sentences are conclusion and should not be mentioned in introduction section.

We thank the reviewer for their comment and have reworked that section.
Method section: where is the statistical analysis section? how did you analyze the results?

We thank the reviewer for their comment and have included the appropriate information.

Lines 264-265 “The experiment was independently performed in triplicate and analyzed via One-Way ANOVA of Dunnett’s test for multiple comparisons using GraphPad Prism 8.”

Lines 275-277 “The fluorescent values were averaged and compared to the control (untreated) wells using unpaired two-tailed t-test. This experiment was performed in triplicate.”
Table 1: What are the sources of isolation for CDC 731-740, MYA-3626, MYA-3627 and etc.?

We thank the reviewer for their question. When able we have listed the isolation source from the supplied. We have added references from their supplier as well.
