# Supplementary material for: Synergistic potential of lopinavir and azole combinational therapy against clinically important Aspergillus species
Source: PLoS One. 2024 Dec 2;19(12):e0314474. doi: 10.1371/journal.pone.0314474 (PMC11611203; doi:10.1371/journal.pone.0314474)
Supplement: Attachment — Submitted filename: Reviewer Replies 091224.docx [file pone.0314474.s003.docx]

Editor Comments:

L113-115: please use Figure 1A, 1B, and 1C.
L231: delete a space before ”Table 1”
L252, 258: condida->conidia

We thank the editor for the comments and have made the necessary changes as follow:

L115: Added (Table 2)

L117: Added “figure 1A-C”

L118: Added “figure 1A-B”

L119: Added “figure 1C”

L120: Added “figure 1A-B”

L231: Could not find, reference, therefore I checked all “Table 1” in document for proper formatting

L 270 and 276: condida→condia (corrected)

L108: Removed “.”

Reviewer Comments:

Reviewer #2: The authors have mostly clarified the queries raised and the modifications suggested. However, looks like no attempts were made to add additional data. At least, the authors can explore whether LPV increases the permeability of the fungus, experimentally, to check whether it is another mechanism facilitating the entry of azoles to execute their function.

### We thank the reviewer for their comment and acknowledge their concern. Our primary focus in this research is LPV’s effect on drug efflux as a mechanism of action. As an impactful mechanism of antifungal resistance occurs through drug efflux. Therefore, once identified as a potent synergizer for resistant isolates we focused in on the mechanism by which it overcame those resistant isolates leading us to study LPV’s effect on efflux.

Reviewer #3: line 87-89: 'Our research revealed that ... observed between POS/ITC and LPV." this sentence should be omitted.

We thank the reviewer for their comment and have made the following corrections. As we wish to keep this statement, we have modified the section for clarity.

L 87-89: “Our work seeks to highlight LPV’s potential role in efflux inhibition, a crucial mechanism for antifungal resistance, facilitating the synergy observed between POS/ITC and LPV.”
