# Supplementary material for: Synergistic potential of lopinavir and azole combinational therapy against clinically important Aspergillus species
Source: PLoS One. 2024 Dec 2;19(12):e0314474. doi: 10.1371/journal.pone.0314474 (PMC11611203; doi:10.1371/journal.pone.0314474)
Supplement: Attachment — Submitted filename: Response to Reviewers 110824.docx [file pone.0314474.s004.docx]

Editors Responses to Questions

L115: Added (Table 2)
L117: Added “figure 1A-C”
L118: Added “figure 1A-B”
L119: Added “figure 1C”
L120: Added “figure 1A-B”
These should be put into parentheses and capitalized at the first character! (This should be applied throughout the text, not used in the required context)

We thank the editor for their comment, and have rechecked the document to reflect this correction.

L139-140: This sentence is too strong and overstated. The data shown by the authors only demonstrated that Rhodamine-6G accumulation associated with LPV treatment. The authors can suggest that LPV inhibit the pump function. Accumulation of azole in the cells treated by LPV was not observed in this study. Please rewrite it to appropriate sentence.

We thank the editor for their comment, and made the following revision to reflect that LPV inhibits efflux machinery utilized to remove toxic dyes,( eg., R6G) and antifungal agents (e.g. azoles). As ABCs transporters have been characterized to remove R6G and play a pivotal role in azole resistance PMID: [32209680](https://pubmed.ncbi.nlm.nih.gov/32209680/).

L138-139: These results demonstrate that LPV can significantly disrupt the function of essential efflux mechanisms that fungi use to expel toxic dyes (e.g. R6G) and antifungal agents (e.g. azoles).

L212-213: the sentence sounds a bit wrong. I think "The cells treated LPV showed 70% more accumulation of Rhodamine-6G than the untreated cells."

We thank the editor for their comment, and made the following revision to reflect the concern.

L210-211: The cells treated with LPV retained 70% more of Rhodamine-6G than the untreated group.
